# Translational Research in Bipolar Disorders

**DOI:** 10.1155/2015/576978

**Published:** 2015-06-14

**Authors:** Rodrigo Machado-Vieira, Benicio N. Frey, Ana C. Andreazza, João Quevedo

**Affiliations:** ^1^Experimental Therapeutics and Pathophysiology Branch, National Institute of Mental Health, NIH, Bethesda, MD 20852, USA; ^2^Department of Psychiatry and Behavioural Neurosciences, McMaster University, Hamilton, ON, Canada L8N 3K7; ^3^Mood Disorders Program and Women's Health Concerns Clinic, St. Joseph's Healthcare, Hamilton, ON, Canada L8N 3K7; ^4^Departments of Pharmacology & Toxicology and Psychiatry, University of Toronto, Toronto, ON, Canada M5S 1A8; ^5^Centre for Addiction and Mental Health, Toronto, ON, Canada M5S 1A8; ^6^Center for Experimental Models in Psychiatry, Department of Psychiatry and Behavioral Sciences, The University of Texas Medical School at Houston, Houston, TX 77054, USA; ^7^Laboratory of Neurosciences, Graduate Program in Health Sciences, Health Sciences Unit, University of Southern Santa Catarina, Criciuma, SC, Brazil

Translational research is at the vanguard of contemporary psychiatry. Its bidirectional continuum (from bench to bedside and vice versa) has become a primary target in the search of personalized treatments in psychiatry. Although the term “translational” has various definitions, its common ground has focused on a better understanding of the pathophysiology of psychiatric disorders as well as on the identification of new diagnostic tests and development of new, improved treatments. In psychiatry, these aims seem more challenging than in other medical areas due to the complexity of brain mechanisms and behaviors.

Several other areas in medicine (e.g., oncology and endocrinology) have successfully decreased the gap between initial drug discovery steps and the subsequent approval of successful therapies for human diseases using translational approaches. In mental health, the use of tools and technologies that are able to positively impact health parameters in psychiatric conditions has been put forward as the ultimate objective of translational neuroscience research, which may tailor clinical practice [1]. Even though several advances have been obtained, this is a key but complex step to pursue, since psychiatric disorders have potential heterogeneous symptomatology, cognitive functioning, and comorbidities as well as having a wide range of genetic and environmental risk factors [2]. Furthermore, recent genome-wide association studies have identified potential candidate genes, but lack of replication by subsequent larger studies and low power have been major limitations in this field.

One example that fits well in this paradigm is bipolar disorder (BD). This is a major mental illness with high prevalence (especially when considering its spectrum), which still has low recovery rates and high rates of treatment-resistant cases, with consequent high morbidity and mortality rates. Despite this major public health issue, existing pharmacological treatments for BD are mostly modifications of older versions and have not been demonstrated to be superior to agents from the same class in terms of efficacy and effectiveness. Except for lithium, all first- and second-line treatments for BD phases and maintenance were first developed and approved for other disorders (e.g., anticonvulsants and atypical antipsychotics). It is noteworthy that the use of lithium has been progressively declining despite the strong evidence base in favor of its use [3, 4].

Another degree of complexity is based on its pathophysiology. This involves dysfunctions at multiple levels and systems with a convergent impact at subcellular downstream cascades that directly regulate cellular resilience and neural plasticity. Some of these molecular targets include central and peripheral stress pathways, inflammation, intracellular signaling cascades, and organelles (mitochondria and endoplasmic reticulum) dysfunction [5]. In this context, some of these newly identified potential treatment targets may represent an advance in the therapeutics of BD, going beyond the so-called “me too” agents. These maybe include modulators at the glutamate and purinergic neurotransmitter systems, intracellular signaling, neuropeptide systems, and others recently tested [6, 7]. These recent developments may also provide new avenues to help neuroscientists explore in more detail the underlying mechanisms involved in the neurobiology of BD using convergent translational approaches [5] and identify new targets for future proof of concept studies.

The use of biomarkers may also play a critical role in this strategy to overcome issues that have limited drug discovery in BD [8, 9]. This includes the use of a new generation of “omics” technologies and the identification of novel mechanisms at genetic, molecular, cellular, cognitive, circuits, and behavioral levels [5]. This approach using biological dimensions at different levels may also help to decipher brain network functioning and validate targets as probes of disease mechanisms. For instance, research on certain candidate genes from genome-wide association studies, such as* ANK3*, a gene involved in the function of voltage gated sodium channels, and* CACNA1C*, a gene encoding a protein which is part of a voltage dependent calcium channel, may represent relevant targets for future functional studies [10] integrated in multiple levels and psychiatric disorders. For instance, genes associated with personality traits and behaviors, such as impulsivity, anhedonia, or suicidality, and involved in circadian rhythm disruption in mood disorders are promising areas to perform studies from molecular to circuit-level.

Even though the previously so expected “holy grail” in psychiatric research seems hard to achieve based on the heterogeneous clinical picture and variable treatment response and polygenic basis of psychiatric disorders, the identification of neural and metabolic (brain/periphery) circuits related to treatment response through longitudinal studies is lacking. Brain imaging studies have also shown cortical and subcortical abnormalities in regions associated with emotional regulation, especially in frontolimbic circuitry; however studies exploring target engagement (e.g., PET receptor occupancy studies) are still scarce and essential to give more consistency when evaluating potential modulators and moderators associated with clinical efficacy in proof of concept trials. Other areas deserving further studies include the neurobiology of cycling, sleep, psychomotor activity studies, and early intervention. Similarly, studies on the role of early stressors/trauma and the role of epigenetics as well as intermediate phenotypes and illness subtypes can benefit from translational approaches in BD research.

Animal and preclinical models also represent valuable tools to further studies underlying neurobiological underpinnings of BD, helping to develop the next generation of strategies using circuit-centered psychiatric dimensions [11]. Findings from clinical neuroscience may be integrated into the next generation of animal models. The main challenge of animal modeling in BD is the inability to develop a model that includes the cycling nature of the illness.

There has been a recent shift in paradigm in psychiatric research. Despite promising perspectives and the appealing search for the “wow factor” when developing new and sophisticated tools and technological machinery with respective technical expertise, these have shown little impact in public health parameters in psychiatric disorders. There is an urgent need for the new generation of clinicians to take a step ahead and be trained to develop skills in neuroscience research, with the same approach for neuroscientists in regard to the study of BD neurobiology and therapeutics, since valuable translational research arises from “clinically relevant” hypotheses. This seems the critical (if not the only) path able to fill the gap between discoveries in basic research and its potential utility and impact in drug development and potential benefit to individuals with mental disorders. Also, a better understanding on the biological basis of the observed variability in treatment response, cognitive dysfunction, neuroprogression, role of comorbidities, and investigation on the treatment-resistant cases are critical aspects to be addressed by the next generation of “clinician-scientists.”

New interesting conceptual frameworks have been recently developed to fill this gap using different units of analysis, such as the NIMH RDoC (Research Domain Criteria) project. This is based on concepts originated from basic behavioral neuroscience. Importantly, this new research framework focuses on the behavior-brain interaction and systems rather than the standard diagnostic classifications [12]. Also, new initiatives such as the NIMH BRAIN initiative [13], which emphasizes the need for sharing large-scale datasets, may help to build the foundation on understanding how the brain works and apply this to the identification of better diagnostic tools and treatments. The development of new neuroscience-based approaches for mental disorders targeted at circuits- and systems-level measurements (e.g., modeling positive and negative affect and anhedonia for depression) may lead to the identification of new targets and more effective treatments.
